# Antioxidant Capacity and the Correlation with Major Phenolic Compounds, Anthocyanin, and Tocopherol Content in Various Extracts from the Wild Edible *Boletus edulis* Mushroom

**DOI:** 10.1155/2013/313905

**Published:** 2012-12-30

**Authors:** Emanuel Vamanu, Sultana Nita

**Affiliations:** ^1^Department of Industrial Biotechnology, Faculty of Biotechnology, University of Agronomical Sciences and Veterinary Medicine Bucharest, 59 Marasti Boulevard, 011464 Bucharest, Romania; ^2^Department of Physical-Chemical Analysis and Quality Control, National Institute for Chemical-Pharmaceutical Research and Development (ICCF Bucharest), 112 Vitan Road, 031299 Bucharest, Romania

## Abstract

*Boletus edulis* is a wild edible mushroom habitually consumed by rural populations. Ethanolic and methanolic extracts was obtained in cold and hot water from dried fruit bodies. The antioxidant activity of freeze-dried extracts from *B. edulis* were investigated using free radicals scavenging activity, reducing power, metal chelating effect, inhibition of lipid peroxidation, and the identification of antioxidant compounds. The levels of different compounds with antioxidant properties were higher in alcoholic extracts compared with aqueous extracts. Rosmarinic acid was the major phenolic compound, it being identified in a concentration between 7 ± 0.23 and 56 ± 0.15 mg/100 g extract. A positive correlation between the content of total phenols, flavonoids, anthocyanins, and tocopherols, and the antioxidant capacity of the extracts was determined. The results showed that the ethanolic extract of Romanian wild mushroom *B. edulis* represents a natural source of functional compounds.

## 1. Introduction

Mushrooms gathered from the wild flora have always been included in the diet of humans for centuries due to their specific taste as well as their medicinal and curative properties. Currently, the existing diversity of mushrooms draws much attention because of the potential of their properties in preventing or treating diseases of the modern world. Recent researches have demonstrated important medicinal properties by *in vitro *assays of wild edible mushrooms, which confirm a plethora of established knowledge gleaned from traditional medicine [1]. These tests have shown a strong antioxidant effect of numerous strains of fungi, which is comparable to that of some classic antioxidants (like vitamins C and E). The most representative properties are the tumor-inhibiting effects and the protective ones, even in everyday domestic consumption, against oxidative processes [2].

These processes are the results of free radicals action, which is also a major cause for the emergence of various inflammatory and degenerative diseases, rising at an alarming rate at present [3]. The most effective treatment, without side effects, aims toward natural products containing antioxidant compounds. A current research topic is the determination of these compounds and the effectiveness of some solvents [4].

*Boletus edulis*, popularly called porcini, is an edible mushroom that grows in forests throughout Romania. It is a basidiomycete fungus that contains a low amount of fat but is rich in minerals, fibers, and proteins. Ergosterol is also present in its composition [5]. The fruit bodies have a high antioxidant capacity and significant alkaloid content [6]. According to some researches, *B. edulis* extracts have a similar amount of phenolic compounds, as does *Agaricus blazei*, of approximately 5.8 mg/g. Thus, it is considered that *B. edulis* has a higher reducing power than *Agaricus bisporus*, *Coprinus comatus*, *Pleurotus eryngii*, or *Pleurotus ostreatus* but is lower than *Pleurotus citrinopileatus* [7].

The main purpose was to determine the antioxidant capacity of ethanolic, methanolic, and cold water and hot water extracts from the fruiting bodies. The researches included the determination of the reducing power, DPPH (2,2-diphenyl-1-picrylhydrazyl) radical scavenging activity, ABTS (2,2′-azinobis (3-ethylbenzothiazoline-6-sulfonic acid) radical scavenging activity, and the inhibition of hydroxyl radicals, nitric oxide, metal chelating effect, and lipid peroxidation. Also, the quantum of components with antioxidant effect was determined.

## 2. Materials and Methods

### 2.1. Chemicals

All chemicals and reagents were purchased from Sigma-Aldrich GmbH (Sternheim, Germany). All the other unlabelled chemicals and reagents were of analytical grade.

### 2.2. Preparation of Samples

Mushrooms were harvested from the forests of Gorj County. Those fungi that were not dirty were chosen, divided into four parts, and dried under a stream of dry air. The drying process took place at a constant temperature of 25°C in an oven, for 15 days, until a constant weight was attained.

### 2.3. Obtaining of Extracts

Dried samples were subjected to extraction with 70% ethanol, 70% methanol, hot water, and cold water. A 10 g sample of the dried mushrooms was extracted using 100 mL solvent (ethanol and methanol) for 24 hours, at 25°C at 150 rpm; 10 g of sample was boiled in 500 mL of water for 30 min. For cold water extraction, a sample (10 g) was extracted by stirring with 100 mL cold water at 4°C for 24 hours [3]. The extract was filtered using a Whatman no. 1 filter paper [8]. The solvents used for extraction were removed using a Buchi R215 rotary vacuum evaporator, with the vacuum controller V-850 and a Multivapor P-6 heating bath for parallel evaporation at 50°C under vacuum [9]. The resulting extracts were lyophilized in an ALPHA 1-2 LD plus freeze dryer (Martin Christ GmbH Gefriertrocknungsanlagen), at −55°C, for 48 hours.

### 2.4. DPPH Radical Scavenging Activity

A quantity consisting of 0.8 mL of 0.2 mM DPPH solution was mixed with a 0.2 mL different concentration of the extracts (0.2–1 mg/mL). The mixture was shaken and left to stand for 30 min. The absorbance was measured at 517 nm using a Helios *λ* spectrophotometer. The DPPH radical scavenging activity (%) was calculated with the following equation: 1 − (*A*_*s*_/*A*_*c*_) × 100, where *A*_*s*_ is the absorbance in the presence of sample and *A*_*c*_ is the absorbance in the absence of sample. Ascorbic acid was used as standard. The IC_50_ value (mg extract/mL), being the inhibitory concentration at which the DPPH scavenging effect is 50%, was obtained [10].

### 2.5. ABTS Radical Scavenging Activity

Radical scavenging assay ABTS radical cations are produced by reacting ABTS (7 mM) and potassium persulfate (2.45 mM) on incubating the mixture at room temperature in darkness for 16 h. The solution thus obtained was further diluted with phosphate buffered saline to give an absorbance of 1.000. Different concentrations of the extracts (0.2–1 mg/mL), 50 *μ*L, were added to 950 *μ*L of the ABTS working solution to give a final volume of 1 mL. The absorbance was recorded immediately at 734 nm with the Helios *λ* spectrophotometer. The percentage of inhibition was calculated with the following equation: % inhibition = [(absorbance of control−Absorbance of test sample)/absorbance control]×100. Ascorbic acid was used as standard. The IC_50_ value (mg extract/mL), being the inhibitory concentration at which the ABTS scavenging effect is 50%, was obtained [11].

### 2.6. Reducing Power

Each extract (in 2.5 mL of ethanol) was mixed with 2.5 mL of 200 mM sodium phosphate buffer (pH 6.6) and 2.5 mL of 1% potassium ferricyanide, and the mixture was incubated at 50°C for of 20 min. Next, 2.5 mL of 10% trichloroacetic acid was added, and the mixture was centrifuged at 3000 g for 10 min. The upper layer (2.5 mL) was mixed with 2.5 mL of deionized water and 0.5 mL of 0.1% ferric chloride. Finally, the absorbance was measured at 700 nm. The extract concentration providing 0.5 of absorbance (IC_50_) was calculated from the graph of absorbance at 700 nm plotted against the extract concentration. Ascorbic acid was used as positive controls [12]. 

### 2.7. Determination of Hydroxyl Radical Scavenging Activity

Quantities consisting of 0.2 mL of 0.1 mM FeSO_4_/0.1 mM EDTA*·*2Na, 0.2 mL 2-deoxyribose (10 mM), 0.2 mL sample (different concentration 0.2–1 mg/mL), and 1.2 mL phosphate buffer (0.1 M; pH 7.4) were mixed. After the addition of 0.2 mL H_2_O_2_ (10 mM), the mixture was incubated at 37°C for 4 h, and the reaction stopped by the addition of a 1 mL trichloroacetic acid (2.8%) solution. Thiobarbituric acid/50 mM NaOH (1%; 1 mL) was then added, and the mixtures were heated at 100°C for 10 min, followed by rapid cooling and measurement of OD_532_ [13]. 

### 2.8. Nitric Oxide Scavenging of Freeze-Dried Extracts

Nitric oxide scavenging activity was measured spectrophotometrically. Sodium nitroprusside (5 mmol/L) in phosphate buffered saline, pH 7.4, was mixed with different concentrations of the extract prepared in ethanol and incubated at 25°C for 30 min. A control without the test compound, but with an equivalent amount of ethanol, was also used. After 30 min, 1.5 mL of the incubated solution was removed and diluted with 1.5 mL of Griess reagent (1% sulphanilamide, 2% phosphoric acid, and 0.1% *N*-1-naphthylethylenediamine dihydrochloride). Absorbance formed during diazotization of the nitrite with sulphanilamide and subsequent coupling with *N*-1-naphthylethylenediamine dihydrochloride was measured at 546 nm, and the percentage scavenging activity was measured with reference to the standard (ascorbic acid). The IC_50_ value (milligram extract/mL) is the inhibitory concentration at which hydroxyl radicals were scavenged by 50% [14].

### 2.9. Ferrous Ion Chelating Assay

1 mL of the sample (0.2–1 mg/mL) was mixed with 3.7 mL of ultrapure water, following which the mixture was reacted with ferrous chloride (2 mmol/L, 0.1 mL) and ferrozine (5 mmol/L, 0.2 mL) for 20 min. The absorbance at 562 nm was determined spectrophotometrically. EDTA was used as a positive control. The chelating activity on the ferrous ion was calculated using the equation following: chelating activity (%) = [(*A*_*b*_ − *A*_*s*_)/*A*_*b*_] × 100, where *A*_*b*_ is the absorbance of the blank without the extract or EDTA and *A*_*s*_ is the absorbance in the presence of the extract or EDTA [15].

### 2.10. Inhibition of Lipid Peroxidation

The reaction mixture contained 1 mL of fowl egg yolk emulsified with phosphate buffer (pH 7.4) to obtain a final concentration of 25 g/L, sample (different concentration of 0.2–1 mg/mL), and 100 *μ*L of 1000 *μ*M FeCl_2_. The mixture was incubated at 37°C for 1 h before being treated with 0.5 mL of freshly prepared 15% trichloroacetic acid (TCA) and 1.0 mL of 1% thiobarbituric acid (TBA). The reaction tubes were further incubated in boiling water bath for 10 min. Once cooled to room temperature, the tubes were centrifuged at 3500 g for 10 min to remove precipitated protein. The absorbance at 532 nm was determined spectrophotometrically. Ascorbic acid was used as positive control. The percentage a inhibition was calculated from the following equation: inhibition (%) = [(*A*_*b*_ − *A*_*s*_)/*A*_*b*_] × 100, where *A*_*b*_ is the absorbance of the blank without the extract or ascorbic acid and *A*_*s*_ is the absorbance in the presence of the extract or ascorbic acid [16].

### 2.11. Determination of Antioxidant Component

#### 2.11.1. Determination of Total Phenolic Content

The content of total phenols was determined by spectrophotometry, using gallic acid as standard, according to the method described by the International Organization for Standardization (ISO) 14502-1. Briefly, an aliquot of the diluted sample extract (1.0 mL) was transferred in duplicate to separate tubes containing a 1/10 dilution of Folin-Ciocalteu's reagent in water (5.0 mL). Then, a sodium carbonate solution (4.0 mL, 7.5% w/v) was added. The tubes were then allowed to stand at room temperature for 60 min before absorbance at 765 nm was measured against water. The content of total phenols was expressed as gallic acid equivalents in g/100 g extract. The concentration of polyphenols in samples was derived from a standard curve of gallic acid ranging from 10 to 50 *μ*g/mL (Pearson's correlation coefficient: *r* ^2^ = 0.9996) [17].

#### 2.11.2. Determination of Total Flavonoids

A sample (0.25 mL of the extracts) was added to a tube containing distilled water (1 mL). Next, 5% NaNO_2_ (0.075 mL), 10% AlCl_3_ (0.075 mL), and 1 M NaOH (0.5 mL) were added sequentially at 0, 5, and 6 min. Finally, the volume of the reacting solution was adjusted to 2.5 mL with double-distilled water. The absorbance of the solution at a wavelength of 410 nm was detected using the Helios *λ* spectrophotometers. Quercetin is a ubiquitous flavonoid, present in many natural extracts, used as standard to quantify the total flavonoid content. Results were expressed in microgram quercetin equivalents/100 g extract [18].

#### 2.11.3. Determination of *β*-Carotene and Lycopene

For *β*-carotene and lycopene determination, the dried ethanolic extract (100 mg) was vigorously shaken with an acetone-hexane mixture (4 : 6, 10 mL) for 1 min and filtered through Whatman no. 1 filter paper. The absorbance of the filtrate was measured at 453, 505, and 663 nm. *β*-Carotene and lycopene content were calculated according to the following equations: lycopene (mg/100 mL) = − 0.0458 × *A*_663_ + 0.372 × *A*_505_ − 0.0806 × *A*_453_; *β*-carotene (mg/100 mL) = 0.216 × *A*_663_ − 0.304 × *A*_505_ + 0.452 × *A*_453_. The results are expressed as mg of carotenoid/g of extract [19].

#### 2.11.4. Determination of Ascorbic Acid

Determination of ascorbic acid was determined by the method described by Barros et al., 2008. Content of ascorbic acid was calculated on the basis of the calibration curve of L-ascorbic acid, and the results were expressed as mg of ascorbic acid/100 g extract [19].

#### 2.11.5. Determination of the Total Quantity of Anthocyanidins

The following solutions were used: ethyl alcohol, hydrochloric acid, sol. 1.5 M, and cyanidin chloride, whereby the solvent was a mixture of 85 : 15 (v/v) of ethyl alcohol and hydrochloric acid 1.5 M. In a measuring bottle of 10 mL, 0.5 g of sample to be analyzed was weighed. Next, the solvent was added up to the mark, and ultrasonation for 15 minutes was performed. Filtering, with the help of Whatman filter paper no. 1, was carried out. As standard solution, the cyanidin chloride with a concentration of 5–15 *μ*g/mL, in solvent was used. The absorption rates of the sample and standard solutions, at spectrophotometer at 546 nm, were determined. The total quantity of anthocyanins (expressed in g of cyanidin chloride/100 g extract) = (*A*_*p*_ × *m*_st_ × *f* × 100)/(*A*_st_ × *m*_*p*_), where: *A*_*p*_ is absorption rate of the sample solution; *m*_*p*_ is mass of the processed sample, in g; *A*_st_ is absorption rate of the standard solution; *m*_st_ is mass of the processed standard solution, in g; *f* is dilution coefficient.

#### 2.11.6. Determination of Polyphenol Carboxylic Acids and Flavones

**Reagents and Equipment**

Methanol HPLC, orthophosphoric acid, ultrapure water, absolute ethanol, high-pressure liquid chromatograph (HPLC) ELITE-LaChrom, with DAD detector, analytic scales KERN 770, and solvent ethanol: water (50 : 50, v/v). Sample solution: in a measuring bottle of 50 mL, we introduced 0.5000 g of sample powder, adding around 40 mL solvent, and performing ultrasonation for 30 minutes at 40°C. Supplements to 50 mL with the same solvent and filters were added as follows. Reference solution was as follows: a mixture of 10 *μ*g/mL: chlorogenic acid, coffee acid, cumaric acid, ferulic acid, luteolin 7-glycoside, rutin, rosemary acid, apigenin 7-glycoside, luteolin, apigenin, quercetin, kaempferol, catechin, myricetin, and pyrogallol.

**Chromatographic Working Conditions**

The chromatographic column from stainless steel comprised the stationary stage of octadecylsilane, (Inertsil ODS-3 250∗4.6 mm∗5 *μ*m); mobile stages: mobile stage A: phosphoric acid/water, pH = 2.5, mobile stage B: methanol; flow rate of the mobile stage: 1.0 mL/min; elution type: with linear composition gradient of the mobile stage; UV detection: *λ* = 330 nm; temperature of column oven: 40°C; injection volume: 20 *μ*L. After the chromatographic system was balanced, the basic line is a straight line, and the reference solution is injected. The differences between successive determinations must be at a maximum of 2%. We injected the test solutions and registered the chromatograms. The content in the compounds of interest was calculated using the formula: compound % = [(*A*_*p*_ × *C*_*e*_) × *A*_*e*_] × (50/*G*) × 100, where *A*_*p*_ is the range of the compound's peak “i” in sample solution; *A*_*e*_ is the range of the compound's peak “i” in the reference solution; *C*_*e*_ is the concentration of compound “i” in the reference solution (mg/mL); *G* is the quantity of processed sample, (mg); 50 is the correction coefficient.

#### 2.11.7. Determination of Tocopherols

**Reagents and Equipment**

Ultrapure water; 2-propanol (R); acetonitrile (HPLC); tetrahydrofuran (R); high-pressure liquid chromatograph (HPLC) ELITE-LaChrom, with DAD detector; analytic scales KERN 770.

**Working Solutions**

In a measuring bottle of 20 mL, exactly 15 mg of sample powder was weighed, adding 10 mL of water and performing ultrasonation for 15 minutes. Supplements to 20 mL with absolute ethyl alcohol, and performing ultrasonation for 5 minutes was carried out. Reference solution: In a measuring bottle of 25 mL, exactly 15 mg of tocopherol was weighed and dissolved in 10 mL of absolute ethyl alcohol and supplements up to the mark with the same solvent; then 2 mL of this solution was diluted at 10 mL, in a measuring bottle with the same solvent.

**Chromatographic Working Conditions**

The chromatographic column from stainless steel comprised the stationary stage octadecylsylane (ZORBAX Eclipse XDB-C8 250∗4.6 mm∗5 *μ*m); mobile stage: methanol : water (95 : 5), v/v; flow rate of the mobile stage: 1.2 mL/min; detective: 280 nm; injection volume: 20 *μ*L. After the chromatographic system was balanced, the basic line was a straight line, and the reference solution was injected. The differences between successive determinations must be at a maximum of 2%. We injected the test solutions and registered the chromatograms. The content in the compounds of interest was calculated using the formula: tocopherol (%) = [(*A*_*p*_ × *C*_*e*_) × *A*_*e*_]×(25/*G*) × 100, where, *A*_*p*_  is the range of the tocopherol peak in the sample solution; *A*_*e*_ is the range of the tocopherol peak in the reference solution; *C*_*e*_ is the tocopherol concentration in the reference solution (mg/mL); *G* is the quantity of processed sample, (mg); and 100 is the correction coefficient.

### 2.12. Statistical Analysis

All the assays for fermentation and antioxidant activity were assessed in triplicate, and the results were expressed as mean ± SD values of the three sets of observations (*P* < 0.05). The mean values and standard deviation were calculated using the Excel program from Microsoft Office 2010 package.

## 3. Results and Discussion

The extractions revealed that the use of the two types of alcohols as solvents leads to a higher extraction efficiency than hot or cold water. The order was as follows: ethanol (25.8 ± 1.69%) > methanol (20.6 ± 1.61%) > hot water (19.3 ± 0.14%) > cold water (17.6 ± 2.3%). Ethanol and methanol have demonstrated a greater capacity for extraction of biomolecules from *B. edulis* fruit bodies, which was also demonstrated for other species of mushrooms, such as *Grifola frondosa* [3].

### 3.1. Scavenging Ability on DPPH Radicals

**Figure 1:**
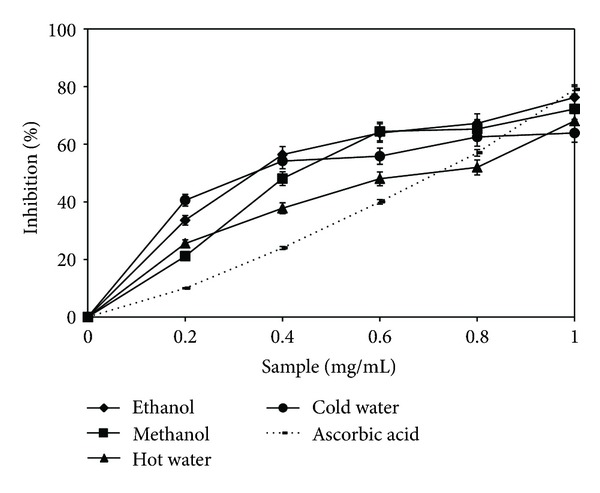
DPPH radical scavenging activity of *Boletus edulis* extracts.

Determination of DPPH scavenging activity represents a rapid method to characterize the antioxidant capacity of extracts against oxidation caused by free radicals. According to this method, the color of the reaction mixture changed from mauve to light yellow, depending on the antioxidant capacity of the four extracts [20]. The ethanolic and methanolic extracts showed a higher capacity of scavenging the DPPH radical than the other two extracts. Maximum values were approximately 72.2%, which was 11.5% higher than those of the hot water extract, and 5.7% higher than those of the cold water extracts, at a concentration of 1 mg/mL (Figure 1). Thus, the values are comparable with those of *A. blazei*, *P. eryngii*, and *C. comatus*. These results are better than those reported by previous researches on extracts from *B. edulis*, which did not use lyophilization for drying the extracts [7]. This difference is evidence to the fact that lyophilization is the most effective process for drying of an extract in order to obtain a maximum antioxidant activity. It is considered that by lyophilization, the highest amount of phenolic compounds is retained, in comparison with other methods of drying an extract [21].

### 3.2. Scavenging Ability on ABTS Radicals

**Figure 2:**
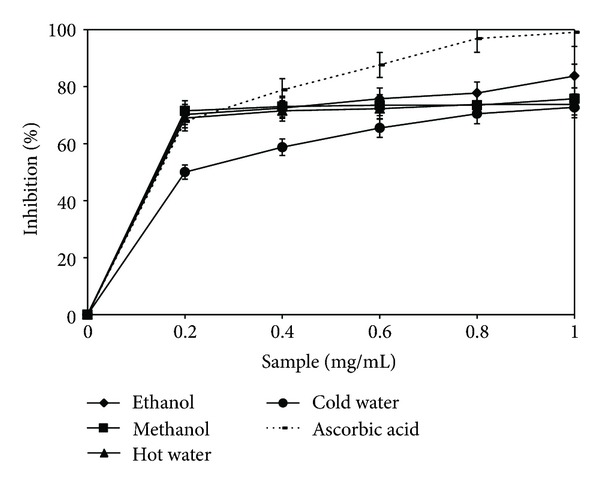
ABTS radical scavenging activity of *Boletus edulis* extracts.

ABTS assay is the second rapid and precise method used for determining antioxidant activity [22]. The scavenging activity of the four extracts, as well as the reference standard, are shown in Figure 2. As noted, the antioxidant activity is directly proportional to the concentration of extracts. Regarding the scavenging capacity of the ABTS radical, the highest activity belongs to the ethanolic extract (83.75%), followed by the methanolic one, with an inhibition of 75.75% at a concentration of 1 mg/mL. In comparison with ascorbic acid, the ethanolic extract activity is 15.4% lower. The noted differences among extracts, as regards the scavenging capacity of ABTS radical compared with DPPH, show the importance of the extraction method for the composition of the four lyophilized extracts. The solvents used determine a different report among substances in the extracts having an antioxidant effect, which is manifested through a different scavenging behavior of free radicals [23].

A significant correlation was noticed between the two methods for determining the antioxidant activity (*R* ^2^ = 0.86) for the methanolic extract, and the highest correlation coefficient was obtained for the ethanolic one (*R* ^2^ = 0.94). A good correlation was also determined for hot water extracts (*R* ^2^ = 0.75) and for cold water (*R* ^2^ = 0.6). The results suggest that the two methods resulted in a relatively similar antioxidant activity of* B. edulis *extracts. Differences between DPPH and ABTS scavenging activity values can be interpreted as being due to the different mechanisms for the two radicals that may explain the results for extracts of the same mushroom in the tests. Quantities of antioxidant molecules that were present in the extracts increased linearly with increasing concentration. The DPPH scavenging assay resulted in obtaining lower values compared with the ABTS scavenging assay, because the DPPH method has more limitations and is characterized by a lower sensitivity. The reaction of DPPH radicals with most biomolecules is slower than ABTS radicals [24, 25].

### 3.3. Reducing Power

**Figure 3:**
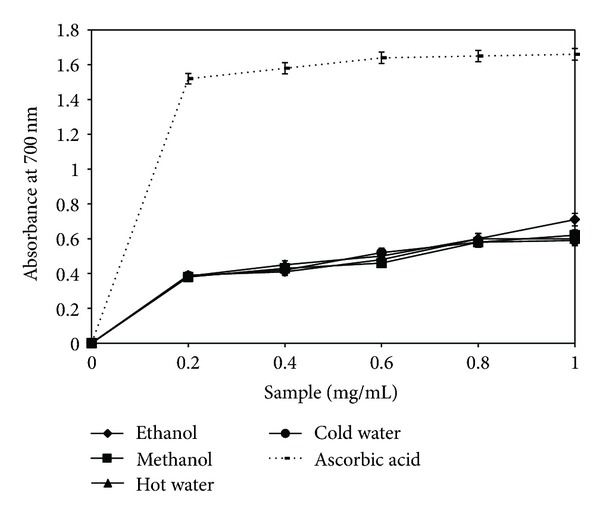
Reducing power of *Boletus edulis* extracts.

Figure 3 shows the reduction power of the four extracts of *B. edulis* and of ascorbic acid as standard, depending on concentration. Following the tests performed, the color of the solution changed from yellow to blue. Thus, it was observed that the extracts' reduction power increased in direct proportion to their concentration. Compared with other medicinal mushrooms (*Ganoderma lucidum*), the analyzed extracts have a similar reduction potential. Compared with the extracts from fungi of the genus *Pleurotus*, the values obtained were, on average, four times higher for concentrations not exceeding 1 mg/mL [16]. The values obtained for the analyzed extracts are very good, with a maximum of 0.71, and the determined order of extracts was ethanol > hot water > cold water. Because it is considered that the reduction power is associated with the presence of reductones, data can lead to the conclusion that reductones might be present in significant quantities, and that they interact with free radicals and block their activity [26].

### 3.4. Metal Chelating Effect

**Figure 4:**
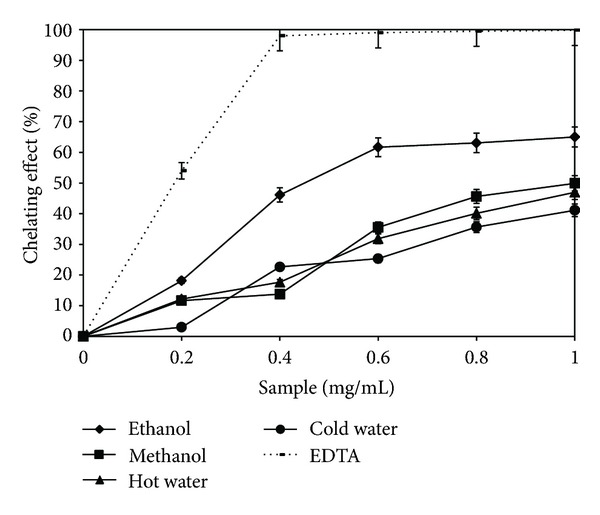
Chelating effect of *Boletus edulis* extracts.

Ferrous iron has the ability to promote the generation of reactive oxygen species, such as superoxide anion radicals, hydroxyl radicals, and nonfree radical species [27]. Also, iron can accelerate lipid peroxidation by the Fenton reaction. By this method, ferrozine forms assemblages with ferrous iron. In the presence of the extract, the process is interrupted, and the violet-reddish color decreases in intensity, thus permitting the spectrophotometric determination of the chelating capacity [28]. Depending on the solvent used, the lyophilized extracts for concentrations of 0.2–1 mg/mL had the following chelating effect: ethanolic extract (18.16–65%) > methanolic extract (11.67–49.9%) > hot water extract (12.17–46.96%) > cold water extract (2.99–41.11%). For a concentration of 1 mg/mL, the standard (EDTA) presented a chelating effect higher, with 32.29 ± 0.97%, compared with the ethanolic extract value (Figure 4).

The results obtained are higher than those of another previous study, which reported similar values for concentrations of 20 mg/mL. Although lyophilization has been used for processing both extracts, the difference was due to the source of the fungus and to the solvent concentration. Thus, a much higher extraction capacity of 70% ethanol in comparison with that of undiluted ethanol was demonstrated [7]. However, similar values were obtained for the ethanolic extract, and higher values for cold water extract from *G. frondosa*, where lyophilized extracts were used [3]. The higher chelating effects of freeze-dried ethanolic extracts from *B. edulis* at low concentrations would be beneficial in food or nutraceutical supplements from natural products [29]. The chelating ability is an important property because it brings about the reduction of the concentration of transition metal that catalyzes lipid peroxidation [30]. The results demonstrate that the efficiency in metal chelation varied with the type and quantity (sample efficiency increases with concentration) of phenolic compounds present in the analyzed lyophilized extract [31].

### 3.5. Inhibition of Lipid Peroxidation

**Figure 5:**
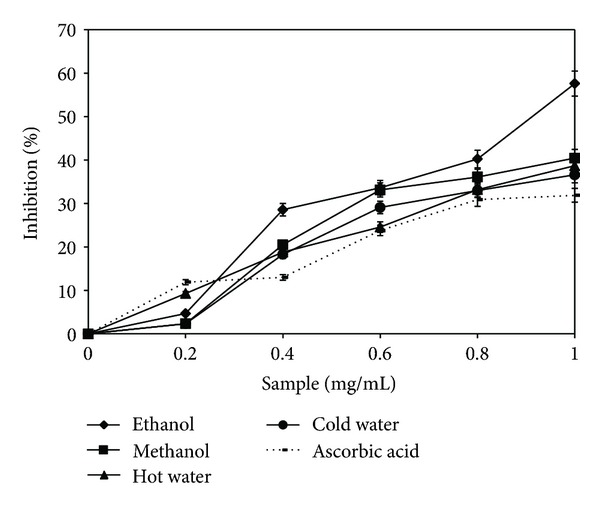
Inhibition of lipid peroxidation of *Boletus edulis* extracts.

 Mushroom extracts constitute a range of natural products with a strong effect against lipid peroxidation. This property is determined by the phenolic component. The first phase of this process is represented by peroxidation of polyunsaturated fatty acid components, which can bring about biological damage at the molecular level. Transition ions (ferrous iron) may generate highly reactive free radicals which can initiate the process of lipid peroxidation [16]. The ability of the *B. edulis* extracts to inhibit peroxidation of phospholipids in egg yolk is presented in Figure 5. In the present study, all four extracts presented inhibition of lipid peroxidation at different levels. At the concentration of 1 mg/mL, the maximum inhibition level of lipid peroxidation is 57.61 ± 0.29% (ethanol extract), and for the ascorbic acid value it was 31.89 ± 1.24% (Figure 5). The inhibition of lipid oxidation of the extracts decreased in the order: ethanolic extract > methanolic extract > hot water extract > cold water extract. The results demonstrated that the ethanolic extract of *B. edulis* has a capacity to inhibit lipid peroxidation significantly more important than the species most used, that of *Agaricus *sp. [32] and *Leucopaxillus giganteus*, regardless of source of nitrogen used for obtaining the mycelium in submerged culture [33]. In contrast, fruit bodies of *Sarcodon imbricatus *and of *L. giganteus* in lyophilized form showed a capacity similar to that of the ethanolic extract, but the sample concentration was of *≈*5 times higher [26].

### 3.6. Scavenging Ability on Nitric Oxide Radical

**Figure 6:**
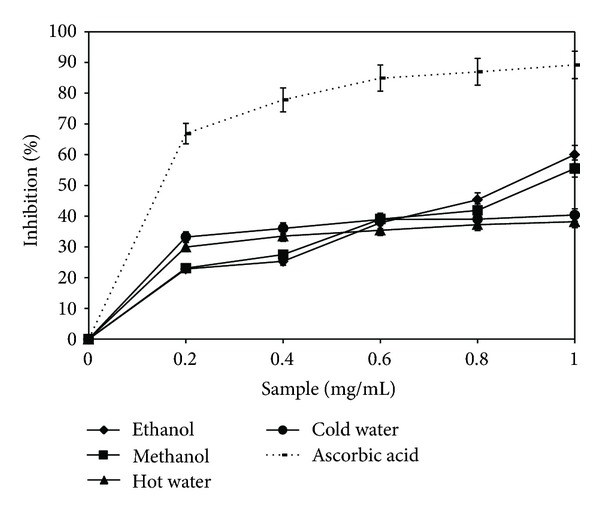
Nitric oxide radical scavenging activity of *Boletus edulis* extracts.

 The nitric oxide assay has been widely used to evaluate the effectiveness of free radicals scavenging on various natural extracts. The generated nitric oxide is a result of the decomposition of sodium nitroprusside in aqueous medium [34]. It could react with oxygen to produce stable products, nitrate and nitrite [35]. Nitric oxide is involved in the regulation of many physiological processes, being associated with numerous human diseases. It is a relatively stable radical and has no negative effects, except in combination with other molecules. It can react with the superoxide radical, generating oxidant peroxynitrite which can induce cell apoptosis [36]. *B. edulis* extracts showed a good scavenging activity on nitric oxide, at a maximum concentration of 1 mg/mL (Figure 6). Thus, the determined order was as follows: ethanol extract (60.5 ± 0.53%) > methanol extract (55.48 ± 0.32%) > cold water extract (40.35 ± 0.19%) > hot water extract (38.16 ± 0.81%). Hot and cold water extracts had lower scavenging abilities, with *≈*50%, than the extracts from *Pleurotus squarrosulus.* However, the methanolic extract showed similar values (*P* < 0.05), on average, 7.5% lower than ethanolic extract [37]. Compared with the extract of *G. lucidum*, the value is ±5.84% lower [38].

### 3.7. Scavenging Ability on Hydroxyl Radical

**Figure 7:**
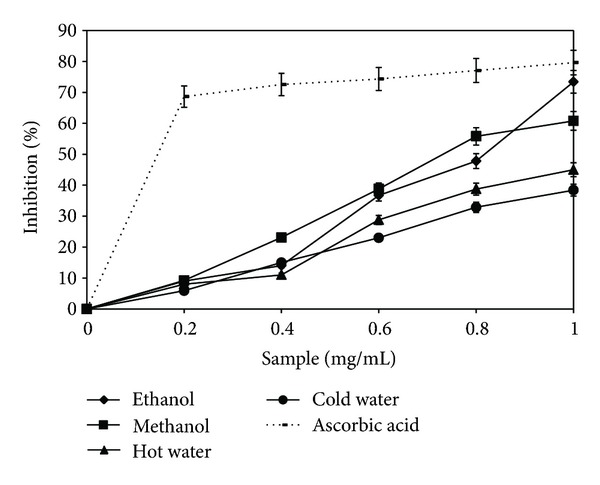
Hydroxyl radical scavenging activity of *Boletus edulis* extracts.

The hydroxyl radical is a highly reactive species of free radicals, involving damage in the biological systems of cell molecules, and being able to form links with DNA nucleotides. By this mechanism, it contributes to the development of carcinogenesis, which is a response of biological systems to the mutation of genetic material in a cell. This is manifested by uncontrolled cell division, which spoils the balance between normal cell proliferation and apoptosis [39]. In this study, ethanolic and methanolic extracts showed a higher scavenging activity than the two aqueous extracts. At 1 mg/mL, the hydroxyl radical activities were 73.4 ± 0.54% and 60.8 ± 0.71% for ethanolic and methanolic extracts, respectively. The other two extracts had a maximum activity of 45 ± 0.43% for hot water extract, and 38.4 ± 1.3% for the cold water extracts (Figure 7). Instead, the standard used (ascorbic acid) had a value of 79.64 ± 1.41% at 1 mg/mL. The results are superior to those of previous researches, which showed similar values of the scavenging ability of the ethanolic extracts and of hot water extracts, at 10 times higher concentrations. Differences are due to the various origins of the biological material and to the extraction process, which in the previous survey was evaporated to dryness at 40°C [7]. The obtained results for the ethanolic extracts are similar to those of a similar extract from *P. citrinopileatus* fruit bodies, of 80.1%, but for concentrations of 20 mg/mL [40].

### 3.8. IC_50_ Values in the Context of Antioxidant Properties

Evaluation of antioxidant properties is summarized in Table 1, and expressed as IC_50_ value. Higher IC_50_ values indicate lower effectiveness in antioxidant properties [3]. The values for the reducing power of the extracts were in a descending order of ethanolic > methanolic > cold water > hot water. For scavenging ability on DPPH radicals, various extracts were effective in the order of ethanolic > hot water > cold water > methanolic extracts; for scavenging ability on ABTS radicals, the order was ethanolic > methanolic > cold water > hot water extracts. For scavenging ability, expressed as IC_50_, on nitric oxide (0.8–1.32 mg/mL) and hydroxyl (0.81–1.58 mg/mL) radical activity, the order was: ethanolic > methanolic > hot water > cold water extracts. The same trend was also determined for the chelating effect on ferrous ions (0.86–1.48 mg/mL), and for inhibition of lipid peroxidation (0.855–2.26 mg/mL).

 Compared with previous studies [7], IC_50_ values were significantly lower for *B. edulis* as regards the power reduction, the determination of antioxidant activity (expressed as scavenging activity on free radicals), ferrous ions' chelating ability, and inhibition of lipid peroxidation. From these results, it was noticed that the ethanolic extract of mushroom species has a significant antioxidant activity against various *in vitro* antioxidant methods [41]. Besides obvious therapeutic effects of the extracts, *B. edulis *mushrooms from Romanian forests might serve as possible nutraceutical foods in human diet, in helping to reduce the oxidative damage. In particular, the ethanolic extracts of this species could be used as a new dietary supplement, with a direct practical application [42].

### 3.9. Antioxidant Components and the Correlation with IC_50_ Values

 The main effect of phenolic compounds is the antioxidant effect. These species are well known, regionally, and are used in folk medicine. Owing to regional differences, each species has a different phenolic profile of its own. However, flavonoids are the most common biomolecules which can be found in plants. Evidence of this has also been found in extracts of edible wild mushrooms. In this case, the order of the quantities of molecules with antioxidant effects depended on the solvent used: ethanol > methanol > hot water > cold water extracts. These determinations are consistent with other recent studies proving the large amount of phenolic compounds' concentration from *B. edulis*, compared with other species: *Lactarius deliciosus, A. bisporus*, *P. ostreatus,* or *Cantharellus cornucopioides*. The differences involved in these studies arise from the different cultivation areas instead of the extraction with different solvents [43].

**Figure 8:**
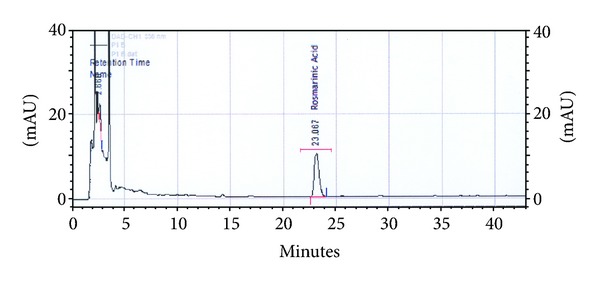
Chromatogram of rosmarinic acid: ethanolic extract.

The profile of phenolic compounds, compared with the corresponding reference reagents, showed only the presence of rosmarinic acid (Figure 8) in the four types of extracts, within the limits of 56 ± 0.23–7 ± 0.15 mg/100 g extract (Table 2). This is the first scientific mention of the presence of this compound in the extract of *B. edulis*. Also, it does not exclude the presence of other phenolic compounds (e.g., homogentisic acid) that is present in most wild edible species, given the difference between total phenol amount and that of rosmarinic acid. Although gallic acid would be another possible phenolic compound present in extracts of this species, it is absent in the composition of biomolecules with antioxidant effect revealed in the present study [43].

 Flavonoid content ranged from 61.42 ± 1.6 to 116.64 ± 4.87 mg quercetin/g extract. Among the tested flavonoids, anthocyanins were the identified biomolecules, with a maximum determined amount in ethanolic extract, of 16.99 ± 0.05 mg cyanidin chloride/100 g extract. The compounds identified in these extracts show that at least a part of the functional compounds in medicinal and/or edible mushroom is due to growing mushrooms on substrates that are high in the functional molecules. To these categories can be added anthocyanins, beta-glucans, selenium, ganoderic acid, triterpenes, or cordycepin [44].

Ascorbic acid was identified in significant quantities (1.3 ± 0.25–5.07 ± 0.14 mg/g extract), comparable with other species, like *Hebeloma sinapizans* (2.8 ± 7.46 mg/g extract) or *Inocybe splendens* (2.61 ± 3.42 mg/g extract) [45]. Alcoholic extracts have a high extraction power of ascorbic acid compared with the aqueous ones. The difference was *≈*50%. Carotenoid compounds were determined in relatively small quantities. Lycopene has been identified only in the ethanol extract, in a measured quantity of 0.024 ± 0.002 mg/100 g extract. The data presented show that lyophilization of the extracts leads to preserving a maximum quantity of the two types of molecules, compared with other procedures of extract processing. These data are confirmed by previous studies which showed that lyophilization is the most efficient way to retain a maximum amount of ascorbic acid and *β*-carotene [46].

**Figure 9:**
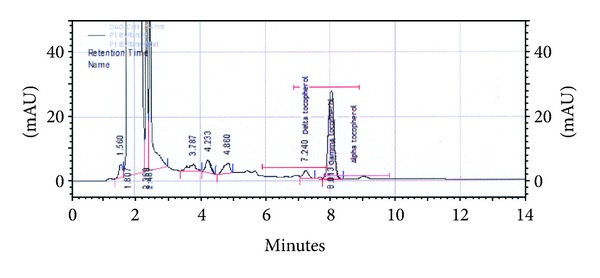
Chromatogram of tocopherols (*α*-, *δ*-, and *γ*-tocopherol): ethanolic extract.

Also, *α*-tocopherol (Figure 9) was not found following the chromatographic analysis, and *δ*-tocopherol is absent in hot and cold water extracts. Differences between the amounts of *δ*-, *γ*-tocopherol in the two alcoholic extracts were significant (Table 2 and Figure 9). The high levels of these two compounds correspond with a higher oxidative activity, which is associated with cardiovascular protection [45].

Antioxidant components in the four extracts from *B. edulis* are responsible for their lower IC_50_ values in antioxidant activity, reducing power, chelating ability on ferrous ions, and inhibition of lipid peroxidation. The total phenols, flavonoids, and *β*-carotene contents of these four extracts exhibited the strongest correlation with IC_50_ values of antioxidant properties, while ascorbic acid and lycopen exhibited weaker correlations. The high amount of total phenolics in ethanolic extracts, compared with aqueous extracts, was demonstrated by the values of the correlation coefficient. The *R* ^2^  values for free radicals scavenging activity ranged between 0.876 and 0.978 for rosmarinic acid, and 0.789 and 0916 for anthocyanins, depending on the type of the extracts and the free radicals; for reducing power, *R* ^2^ was 0.726–0.879 for rosmarinic acid and 0.691–0.742 for anthocyanins; for lipid peroxidation, *R* ^2^ was 0.965–0.907 for rosmarinic acid, and 0.587–0.669 for flavonoids; for chelating ability, *R* ^2^ was 0.765–0.834 for rosmarinic acid and 0.467–0.678 for anthocyanins. The two types of tocopherols determined a significant correlation with the chelating ability (0.754–0.815), and the lipid peroxidation inhibition (0.803–0.869) but lower for free radicals scavenging activity. The correlation coefficient between *β*-carotene contents of extracts and antioxidant activity was relatively low (0.435–0.519).

## 4. Conclusions

Consequently, the possible differences in antioxidant activity demonstrated, in addition, by the *R* ^2^ values between different studies may be due to different phenolic profile of the biomolecules that determine this property, mainly of phenolic compounds. There is a high degree of variation among the different categories of biomolecules with antioxidant effect in each type of extract, or determined by the provenience source of the biological material subjected to extraction. The variation of the antioxidant activity can be also attributed to the solvent, because it depends on its type and polarity and on the procedures for isolation and quantification. The favorable correlation of rosmarinic acid, anthocyanins, and tocopherols with all antioxidant scavenging assays could mean that other compound classes with important roles are not present. Thus, the evaluation of compounds with antioxidant profiles from Romania revealed the presence of rosmarinic acid, which represents a novelty in the study of fungi, because of its important biological effects.

## Figures and Tables

**Table 1:** IC_50_ values of various extracts from *Boletus edulis*.

	IC_50_ (mg/mL)					
	Ethanolic	Methanolic	Hot water	Cold water	Ascorbic acid	EDTA
Scavenging ability on DPPH radicals	0.62 ± 0.12	0.73 ± 0.07	0.57 ± 0.04	0.66 ± 0.21	0.09 ± 0.03	—
Scavenging ability on ABTS anion	0.3 ± 0.09	0.42 ± 0.07	0.47 ± 0.02	0.45 ± 0.11	0.24 ± 0.03	—
Reducing power	0.31 ± 0.04	0.34 ± 0.1	0.49 ± 0.09	0.43 ± 0.36	0.86 ± 0.05	—
Scavenging ability on hydroxyl radical	0.81 ± 0.22	0.86 ± 0.31	1.37 ± 0.45	1.58 ± 0.38	0.07 ± 0.02	—
Scavenging ability on nitric oxide	0.8 ± 0.1	0.98 ± 0.24	0.98 ± 0.28	1.32 ± 0.32	0.08 ± 0.01	—
Chelating effect	0.86 ± 0.17	1.07 ± 0.24	1.34 ± 0.3	1.48 ± 0.07	—	0.048
Inhibition of lipid peroxidation	0.855 ± 0.41	1.17 ± 0.11	1.58 ± 0.15	2.26 ± 0.31	4.9 ± 0.57	—

**Table 2:** Antioxidant contents of extracts from *Boletus edulis. *

Antioxidant components	Extract			
	Ethanolic	Methanolic	Hot water	Cold water
Total phenols (mg gallic acid/g extract)	21.32 ± 3.78	18.96 ± 1.74	17.22 ± 2.08	15.98 ± 0.74
Rosmarinic acid (mg/100 g extract)	56 ± 0.23	28 ± 0.19	18 ± 0.2	7 ± 0.15
Flavonoids (mg quercetina/g extract)	116.64 ± 4.87	97.5 ± 1.44	82.85 ± 2.21	61.42 ± 1.6
Anthocyanins(mg cyanidin chloride/100 g extract)	16.99 ± 0.05	11.98 ± 0.11	10.49 ± 0.32	6.98 ± 0.05
Ascorbic acid (mg/g extract)	5.07 ± 0.14	3.7 ± 0.06	1.86 ± 0.34	1.3 ± 0.25
*β*-Carotene (mg/100 g extract)	0.087 ± 0.002	0.004 ± 0.003	0.0078 ± 0.001	0.0074 ± 0.002
Lycopene (mg/100 g extract)	0.024 ± 0.002	—	—	—
*α*-Tocopherol (mg/100 g extract)	—	—	—	—
*δ*-Tocopherol (mg/100 g extract)	0.075 ± 0.001	0.006 ± 0.002	—	—
*γ*-Tocopherol (mg/100 g extract)	0.711 ± 0.02	0.024 ± 0.001	0.008 ± 0.005	0.007 ± 0.001
